# Physico-Chemical Conversion of Lignocellulose: Inhibitor Effects and Detoxification Strategies: A Mini Review

**DOI:** 10.3390/molecules23020309

**Published:** 2018-02-01

**Authors:** Daehwan Kim

**Affiliations:** Laboratory of Renewable Resources Engineering, Department of Agricultural and Biological Engineering, Purdue University, West Lafayette, IN 47907, USA; kim1535@purdue.edu; Tel.: +1-765-637-8603

**Keywords:** biofuels, ethanol, lignocellulose, pretreatment, hydrolysis, fermentation, inhibitors, detoxifications, phenols

## Abstract

A pretreatment of lignocellulosic biomass to produce biofuels, polymers, and other chemicals plays a vital role in the biochemical conversion process toward disrupting the closely associated structures of the cellulose-hemicellulose-lignin molecules. Various pretreatment steps alter the chemical/physical structure of lignocellulosic materials by solubilizing hemicellulose and/or lignin, decreasing the particle sizes of substrate and the crystalline portions of cellulose, and increasing the surface area of biomass. These modifications enhance the hydrolysis of cellulose by increasing accessibilities of acids or enzymes onto the surface of cellulose. However, lignocellulose-derived byproducts, which can inhibit and/or deactivate enzyme and microbial biocatalysts, are formed, including furan derivatives, lignin-derived phenolics, and carboxylic acids. These generation of compounds during pretreatment with inhibitory effects can lead to negative effects on subsequent steps in sugar flat-form processes. A number of physico-chemical pretreatment methods such as steam explosion, ammonia fiber explosion (AFEX), and liquid hot water (LHW) have been suggested and developed for minimizing formation of inhibitory compounds and alleviating their effects on ethanol production processes. This work reviews the physico-chemical pretreatment methods used for various biomass sources, formation of lignocellulose-derived inhibitors, and their contributions to enzymatic hydrolysis and microbial activities. Furthermore, we provide an overview of the current strategies to alleviate inhibitory compounds present in the hydrolysates or slurries.

## 1. Introduction

Lignocellulosic biomass ethanol production, commonly referred to as Second Generation biofuels, using agricultural residues, forest residues, energy feedstocks, municipalities, and other waste crop solids is considered as a promising alternative energy source in order to minimize reliance on limited fossil sources, greenhouse gas emissions, and environmental pollutions [1,2,3,4]. A plentiful availability of lignocellulosic materials also encourages the production of numerous commodities and applications to foods, chemicals, textiles, and biofuel sources [5,6,7]. In response to ever increasing global concern as well as major interest, a large number of research groups, companies, and nations involved in renewable and sustainable energy have begun investigating lignocellulosic materials. However, lignocellulose has a complex molecular structure, with tangled chains of cellulose, hemicellulose, and lignin, requiring a pretreatment step in order to improve a fermentable sugar yield for subsequent production of value added molecules, such as, ethanol, at low cost. A physico-chemical pretreatment step is commonly utilized to disrupt a crystalline structure of lignocellulose molecules, which is capable of improving bioconversion of lignocellulosic feedstocks to biorefineries. Various methods for the pretreatment process have been developed over the last three decades that includes physical (milling and grinding), chemical (acid, alkaline, and other organic solvents), physio-chemical (hot water, ammonia fiber explosion, and steam explosion), and biological (fungi, bacteria, and microalgae) methods [8,9,10,11]. However, additional lignocellulose-derived compounds, can be generated during pretreatment that have an inhibitory effect the on enzymatic and process performance, which results in decreased fermentable sugar yields and negatively impacted subsequent ethanol productions. These inhibition issues increase total ethanol production cost since more enzyme is required for hydrolysis. This inhibition effect is considered as the principal bottleneck for practical bioethanol production from lignocellulosic materials [12,13]. The aim of this review is to highlight the recent investigatory effects on physico-chemical pretreatment methods, such as steam explosion, ammonia fiber explosion, and liquid hot water, their formation of lignocellulosic-derived-inhibitory compounds (sugar-derived compounds, lignin-derived phenolics, oligosaccharides, and weak organic acids), and current strategies to alleviate the negative effects of these inhibitors on enzymatic hydrolysis and microbial activities.

## 2. Key Factors for Effective Pretreatment

### 2.1. Structure of Lignocellulosic Biomass

Lignocellulosic biomass consists mainly of cellulose, hemicellulose, lignin, small amounts of extractives, and ash (Table 1). Cellulose, which accounts for approximately 40% of the total dry weight of the lignocellulose with multiple polysaccharide linear chains, is embedded in a resistant structure of hemicellulose and strengthened by hydrogen bonds. Cellulose/hemicellulose chains are protected by lignin and acetyl compounds, linked to the macrostructure by covalent bonds. In response to the recalcitrance of lignocellulosic biomass, pretreatment processes have been studied to break down the intricate structure of plant macrostructure, and exposed polysaccharides can be liberated to monomeric sugars via following the hydrolysis step with acid, alkali, or enzyme.

It is recognized that lignocellulose recalcitrance and enzymatic saccharification are highly related to the crystallinity of polysaccharide [31], the degree of polymerization (DP) [32], the lignin content [33,34,35], and the surface areas (porosity) [36]. The brief biomass structural/chemical properties and their recalcitrant effects on pretreatment and enzymatic hydrolysis are summarized in Table 2. Therefore, an effective pretreatment process considers the properties and characteristics of lignocellulosic materials to improve the cellulose and/or hemicellulose conversion yields. Major factors for an effective pretreatment has several objectives, including: (1) decrease the crystallization and increase the surface area of cellulose for enzymatic digestion; (2) solubilize hemicellulose and/or lignin; (3) avoid the loss of sugars; (4) minimize the formation of undesirable lignocellulose-derived inhibitors; and (5) minimize the energy and capital costs.

### 2.2. Cellulose Crystallinity and Degree of Polymerization (DP)

The crystalline portion of cellulose is thought to be one of the main parameters for an efficient hydrolysis because the high level of crystalline structure is less susceptible to degradation by enzymatic methods than the amorphous portion of cellulose [37,38]. The reduction of crystallinity through a pretreatment process has been considered as a determining cellulose conversion yield and hydrolysis rate at low enzyme loadings. Other works suggest that enzyme binding kinetics and an enzymatic digestion efficiency are highly correlated to the accessible surface area of cellulose [39,40]. Cellulose has both internal and external surface area; a small vein structure (capillary tube) of cellulose which is related to internal surface area, and a small particle size (increased external surface area) which is able to improve conversion yield by allowing more enzyme access onto the surface of cellulose. For example, when the particle size of liquid hot water pretreated mixed hardwood was reduced from 3 mm to 2 mm, cellulose conversion to glucose was improved up to 50% [41]. Even with the use of un-pretreated substrate at the similar experimental conditions, decreasing particle size (<0.3 mm) increased the glucose yield from 4.6% to 14% [41]. Furthermore, cellulose degree of polymerization (DP) is considered to be another major contributor that affects cellulose hydrolysis. Low DP biomass has more reducing ends that are more susceptible to be broken down to monomeric molecules than those from the high DP. Pretreatment at a high severity or a high enzyme loading is capable of reducing the cellulose DP [42,43,44,45].

### 2.3. Lignin

The presence of lignin in lignocellulosic feedstock contributes towards the closeness and integrity of the lignocellulose structure by acting as solid adhesives to cellulose and hemicellulose. Lignin, which contains diverse phenolic acids including *p*-coumaryl, coniferyl, guaiacyl, syringyl and sinaphy, is widely recognized as not only an important indocile molecule, but also one of the dominant compounds that can release various inhibitory byproducts during pretreatment step [36,46,47]. Since lignin-derived compounds are among the most influential inhibitors in enzymatic reaction and microbial fermentation, delignification process may be necessary for improving the cellulose conversion [48,49,50]. Changes in lignin composition can depend on the severity of the pretreatment of the biomass; lignin content was shown to relatively increase as the severity of the pretreatment increased, likely due to the de-polymerization/re-polymerization of the lignin structure [51,52]. Lignin can also cause direct and/or indirect inhibitory effects on enzymes catalysis. The non-productive bindings of cellulase/hemicellulase enzymes to lignin molecules remarkably decrease the enzyme activities, possibly due to lignin-enzyme hydrophobic interactions [33,50,53]. It has been previously reported that the recovered lignin from liquid hot water pretreated hardwood can adsorb the cellulase enzyme by up to 60% when incubated for 1.5 h at 25 °C [34]. Furthermore, the modified lignin via pretreatment has a heterogeneous condensed structure, which can affect cellulase enzyme more severely than the lignin isolated from non-pretreated biomass [33,34].

### 2.4. Hemicellulose

Hemicellulose, consists of five and six different carbon sugars such as xylose, arabinose, galactose, and mannose, is more liable to be hydrolyzed than cellulose. However, it primarily protects the cellulose fiber from the enzymatic attacks. One of the main goals of pretreatment is to solubilize hemicellulose, the removal of hemicellulose expects more enzyme accessibility on to the cellulose with the larger pore volumes [54]. Hemicellulose polymers have broadly linked with acetyl groups, and they can hamper the appropriate enzyme binding on cellulose. Generally, a pretreatment with acid chemicals releases the acetyl group from hemicellulose that has inter-linkages in the xylan backbone [55,56,57]. In order to minimize the acetyl group inhibitions from lignocellulose, deacetylation process with acetic acid was proposed, however, enzyme inhibition still required further investigated [12,56,58].

## 3. Physico-Chemical Pretreatment

Steam explosion is one of the most extensively studied and developed pretreatment methods for physico-chemical conversion of lignocellulose feedstock. Steam explosion pretreatment causes explosive decomposition in the biomass due to an abrupt pressure change that could physically breakdown fiber structures. The main advantages of steam explosion are cost effectiveness, decreased environmental influence, lower energy requirement, and little to no chemical usage [44,59]. Compared to the conventional mechanical process, steam explosion method requires a 70% lower energy consumption to obtain the same particle size of substrate [1]. This method can also be applied to a practical plant scale, for example, industrial steam explosion currently manufactured by Canada Sunopta Company, they use 500 kg/h continuous steam explosion facilities. During the steam explosion process, chipped materials are treated at a high temperature range of 160–260 °C for a few seconds to a few minutes in the saturated steam. The steam is able to expand the cell wall of the polysaccharide fiber, which contributes toward increasing enzyme accessibility to cellulose, by exposing internal cellulose surface as well as hydrolyzing acetyl groups of hemicellulose to acetic acid (autohydrolysis) [60,61,62,63]. The physical forces can have significant effects, causing hemicellulose solubilization and structure change in lignin, allowing for more effective enzyme binding on to the cellulose. The major parameters that affect steam pretreatment efficacy include biomass origin, particle size, temperature, residence time, and moisture content [64,65,66]. Commonly, it is more effective to hydrolyze cellulose from agricultural residue (straw, sugar cane, and bast fibers) and hardwood (beech wood, willow, and popular) to monomeric sugars than softwood (spruce, Douglas fir, and pine), because of lower acetyl groups in hemicellulose composition [1,44]. The high contents of xylan and acetyl group in hardwood composition can provide a high level of acetylation with *O*-acetyl-4-*O*-methylglucuronoxylan. However, the xylan in softwood mainly include α-l-arabinofuranose linked by α-1,3-glycosidic bonds which are not acetylated [67]. Supplementation of low concentrations of sulfuric acid, SO_2_, or CO_2_ prior to stream explosion have been proposed to improve the pretreatment process that may result in reduction of reaction time and temperature, and increase of conversion yield [68]. Low-pressure steam explosion coupled with ethanol extraction were available to achieve > 30 g/L of sugars and 80% hemicellulose recovery from wheat straw [69]. However, this process also has drawbacks of requiring acid addition which may require additional steps for neutralization and/or detoxification of the hydrolysates. It also generates undesirable inhibitory byproducts that may interfere with subsequent processes [17].

Compared with steam explosion, liquid hot water (LHW) pretreatment is performed in the liquid state with water at various temperatures (160–240 °C), instead of steam [70]. LHW process primarily solubilizes hemicellulose and/or lignin, and exposes internal cellulose contents which are capable of increasing enzyme accessibility to cellulose. In addition, hemicellulose derived-sugars mostly exist as oligomers forms in the liquid fraction that result in minimal formation of undesirable degraded compounds [17,71]. This method provides few advantages compared to other pretreatment methods: (1) it requires no additional catalysts or chemicals; (2) it conducts at relatively moderate temperature; (3) it minimizes sugar loss and formation of inhibitory compounds; and (4) it is cost effective [8,10,72]. The LHW pretreatment disrupts hemicellulose linkages and releases various liberating acids, which play a role in catalysts and hydrolyze oligosaccharides and monomeric sugars to degraded aldehydes forms (i.e., furfural from five carbon sugars and HMF from six carbon sugars). However, during LHW pretreatment, autocatalytic generation of potent inhibitory molecules also occurs [73,74]. The pH control during treatment minimized inhibitory compound formation and subsequent enzyme digestion of cellulose from corn stover to glucose reached up to 90% conversion yield; LHW pretreatment of corn fiber at 160 °C for 20 min and a pH above 4.0 solubilized 50% of the corn fiber and dissolved soluble oligosaccharides up to 80% [71]. While LHW method offers higher C5 sugar recovery and lower sugar-derived inhibitory compounds, it is yet not economically practical at industrial scale due to high energy cost and water demand when compared with steam explosion.

Ammonia fiber explosion (AFEX) is similar to steam explosion with the biomass being liquid ammonia at high temperature (90–100 °C) for several minutes (30–60 min). High pressure and temperature allow for sudden expansion of the ammonia that contributes to swelling and physical breakdown of biomass structure and partially reduces the crystalline portion of cellulose and lignin [75]. This has several advantages: (1) no production of inhibitors; (2) high recovery of sugars (up to 99%); (3) no additional steps for reduction of particle size before pretreatment or water wash after process; (4) ammonia can be recycled; and (5) nitrogen source is not required for subsequent microbial fermentation performances, since liquid ammonia in process can be provided [76,77,78,79]. However, AFEX method is more beneficial to herbaceous, agricultural crops, and lower lignin feedstocks than woody biomass (high lignin composition) because only slight amounts of hemicellulose and lignin are dissolved during the pretreatment [80]. For instance, enzymatic hydrolysis of the AFEX pretreated corn silage and whole corn obtained 1.5–3 times higher conversion yields than those from the un-pretreated samples, but this effect did not translate to high-lignin biomass [81]. AFEX process can be optimized with experimental conditions including ammonia concentration, moisture content, pressure, and reaction time. Further study done by Teymouri et al. [77] showed that cellulose and hemicellulose in corn stover can be enzymatically hydrolyzed to monomeric sugars with the conversion yield of >90%. More recent works demonstrated that the AFEX pretreatment could be applied to simultaneous saccharification and co-fermentation (SSCF) with corn stover and switchgrass, the hydrolysates were fermented through *E. coli* and recombinant *S. cerevisiae* (ferment both C_5_ and C_6_ sugars), respectively [82,83].

## 4. Formation of Inhibitory Compounds from Physico-Chemical Pretreatment

**Figure 1:**
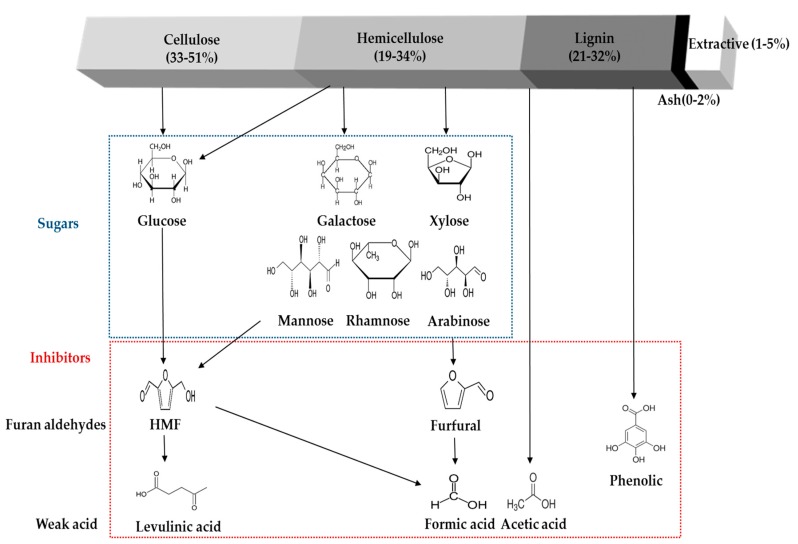
The average chemical composition of lignocellulosic materials and brief scheme of main inhibitory compounds formation.

While many pretreatments have been suggested and investigated to enhance the total sugar recovery and the value of the subsequent chemicals produced, some crucial problems are still hamper the effective enzymatic hydrolysis of cellulosic materials [46,84,85,86] and fermentation process [19,87,88,89]. These pretreatment processes allow for the removal of most of the hemicellulose and partially solubilize the lignin, both of which cause an increase the enzyme accessibilities to the exposed cellulose which can result in the enhancement of conversion yield [90,91]. However, undesired lignocellulose-derived compounds can also be released during the pretreatment, such as furans (furfural and 5-hydroxymethylfurfural), organic acids (acetate, formic acid, and levulinic acid), phenolic compounds, lignocellulose extractives (acidic raw material resin and tannic acid), and other soluble mono-, oligomeric sugars. The main lignocellulose-derived compounds are briefly presented in the Figure 1. The inhibitory molecules present in the pretreated hydrolystes could be categorized into four groups, (1) phenolic compounds: dominantly degraded from lignin content and other aromatic compounds from the biomass; (2) furan aldehydes: primarily present in the pretreated hydrolysate liquid fraction that generated from the sugar (pentose and hexose) degradation; (3) carboxylic acids: degradation byproducts from mainly hemicellulose and furan derivatives; and (4) soluble sugars: hydrolyzed intermediate and end products of the lignocellulosic materials.

The formation of degradation molecules from lignocellulosic materials strongly depends on the type of raw material (chemical composition, solid concentration, and solid property), pretreatment method (physical, acid-based, alkaline-based, hydrothermal, oxidative, alternative solvent, and biological), and pretreatment severity (temperature, pressure, pH, redox reaction, and addition of catalyst) [12,13,66,86,87,92,93,94]. While many pretreatment studies have been performed, the optimal method for minimizing inhibitory molecules still remains to be investigated. 

Cara et al. [27] tested the ethanol production via stream explosion pretreated olive tree pruning at the various temperature range 190–240 °C with impregnation water or sulphuric acid. Each experimental run generated various concentrations of inhibitors that commonly increased when the pretreatment performed at the harsh conditions (Table 3). Similar works also observed that the formation of inhibitory compounds from steam pretreated wheat straw and hardwood were significantly affected by temperature, residence time, substrate size, and sulfuric acid concentration (Table 3) [61,63]. There have many investigations to identify liquid hot water pretreatment of high-lignin biomasses such as hardwood, corn stover, and sugarcane bagasse. LHW pretreatment of maple (23% *w*/*w*, g dry solid/g total) at 200 °C for 20 min released sugar oligomers, acetic acid, furan derivatives, and phenolic compounds (Table 3); ethyl acetate extracted phenolics from LHW-pretreated maple had remarkable inhibitory effects on the pure cellulose (Solka Floc) conversion to glucose by decreasing 20% lower yield than control (no phenolics) [86]. When hardwood at 15% (*w*/*w*, g dry solid/g total) was LHW pretreated at 195 °C for 10 min, the major inhibitors were phenolics (5.9 g/L) and xylo-oligomers (56 g/L) [87]. In this work, they also identified that the washate solution of hydrolysate contained various soluble inhibitors and they could significantly decrease pure cellulose (Avicel) hydrolysis to glucose by 20–30%, which had an approximately 60–70% lower yield when compared to control test with just buffer (88% yield). The formation of inhibitors from LHW-pretreated sugarcane bagasse depended on the severity factor (mainly temperature and residence time); a relatively low severity factor (log *R*_0_ = 3.83) released more oligosaccharides and created less degradation compounds (furans and phenolics) whereas LHW at severity factor of log *R*_0_ = 4.42 produced higher amounts monosaccharides and fractionated molecules (Table 3) [95]. The pretreatment conditions with different pretreatment temperature (T) and reaction time (t) were evaluated as a severity factor, log *R*_0_ (*R*_0_ = *t* × exp((T − 100)/ω)) where ω denotes an activation energy for pretreatment [33,34,96]. The similar observation was confirmed with LHW-pretreated corn stover, which helped to demonstrate cellulase inhibition by lignocellulose-derived products [19,84].

In contrast with steam explosion and LHW methods, AFEX pretreatment generates little to no inhibitory compounds, as only small portions of feedstock solids were solubilized and did not contribute to the production degradation compounds from hemicellulose and lignin [98,99]. The study of Balan et al. [97] identified that the pretreated poplar had degradation compounds, including, phenolics (2.1 mg/g solids), furans (8.6 µg/g solids), and aliphatic acid (1.8 µg/g solids).

## 5. Pretreatment-Derived Inhibitors of Enzymatic Catalysts and Microbial Fermentations

### 5.1. Phenolic Compounds

Multiple phenolic compounds are produced by the degradation of lignin during pretreatment of biomass that are relative to molecular weights, polarities, and side chains. Several aromatic molecules which exist in the lignocellulose may also be released as extractives during sugar degradation. Phenols have been shown to be strong inhibitors to cellulolytic enzyme. For instance, the presence of vanillin at 10 mg/mL decreased the cellulose conversion of lignin-free cellulose (Avicel) by 26%, which was almost a half conversion yield when compared to the control (53%, without vanillin) [39]. It is also found that *p*-courmaric acid and ferulic acid were shown to reduce the cellulose conversion to glucose by around 30% and 16%, respectively [39]. Furthermore, phenolics recovered from the pretreated biomass had a much greater impact on enzyme performance. Michelin et al. [95] observed that phenolics from liquid hot water pretreated sugarcane bagasse (log *R*_0_ = 3.83, 3.5 mg phenolic/mg protein enzyme) led to a 20% lower cellulose (Solka Floc) conversion compared to a control while the phenolic compounds recovered at higher severity condition (log *R*_0_ = 4.42, 6.2 mg phenolic/mg protein enzyme) resulted in a 45% lower yield [95]. Another study showed that phenolics obtained from the liquid hot water pretreated hardwood (log *R*_0_ = 4.25, 2 mg phenolic/mg protein enzyme) decreased conversion yield of by about 50% when they incubated with cocktailed enzymes hydrolysis of Spezyme CP and Novozyme 188 [86]. Cellulase adsorption to the hydroxyl groups derived from phenolic compounds and lignin derivatives also contributed to the inhibitory effects [39].

Some researches indicated that the phenolic compounds are more toxic than other potent inhibitory molecules (furan aldehydes, weak acids and other degraded-by products), even at lower concentrations, since their low molecular weight (MW) allow them able to penetrate cell membranes and damage internal structures, as well as causing changes in the morphology of cells [13,88,89,94]. Ezeji et al. [100] reported that ferulic acid and *p*-coumaric acid were found to be among the most toxic of the phenolic acids tested for a *Clostridium beijerinckii* BA101 bacteria strain, with the presence of 1 g/L of these compounds inhibiting the cell growth by up to 74%. Another study investigating the toxicity level of ferulic acid (1.8 mM) and *p*-coumaric acid (9.7 mM) on a *Saccharomyces cerevisiae* yeast strain reported inhibition of cell growth by up to 80% when compared to the growth without acids [101]. Phenolics can also increase the fluidity of the cell membrane, possibly causing intracellular potassium levels to drop significantly [102]. Furthermore, phenolic compounds are able to promote a loss of integrity in biological membranes, consequently decreases cell growth and further sugar assimilation as well as can cause breakdown of DNA, resulting in the inhibition of RNA and protein synthesis [94,102].

### 5.2. Furan Derivatives

Furfural and 5-hydroxymethylfurfural (HMF) are furan derivative degradation products of pentoses and hexoses, commonly found in hydrolysates. These molecules are not known to significantly inhibit enzymes performances; however, they can negatively affect the microbial fermentation of the treated materials by inhibiting cell growth and sugar uptake rate, subsequently decreasing ethanol production rate [88,89]. Cellulase activity was affected by acetic acid and furfural at the concentrations up to 13 g/L and 4 g/L, respectively [86]. While furan inhibition could delay the total fermentation process by increasing the lag phase of cells, it commonly did not have remarkable effects on the total ethanol yield in *S. cerevisiae* and *Zymomonas mobilis* [11]. In addition, increasing initial cell inoculum of *S. cerevisiae* could reduce the furfural inhibition on the fermentation [103]. However, high concentration of furans or combined with other components medium (mixed with acetic acid, furfural, and lignin derivatives) could be detrimental to microbial growth and fermentation response. For instance, there was no effect on the cell growth of *Scheffersomyces stipitis* at 0.5 g/L of furfural while furfural at 2 g/L was harmful to cell growth [104]. Similarly, during ethanol production from wheat straw hydrolysates by *S. stipitis*, the presence of furfural at 0.25 g/L did not affect the cell growth and ethanol production, while furfural at an elevated concentration of 1.5 g/L inhibited ethanol yield and productivity by 90.4% and 85.1%, respectively [105]. Notably, they also observed a synergetic inhibition between acetic acid, furfural, and lignin derivatives which resulted in decrease yield and productivity than the combined inhibition of singular compounds [105].

Furan derivatives also showed negative effects on the microbe kinetics, affecting metabolisms, cell wall formation, and DNA, plasmid, RNA and/or protein synthesis [106,107,108]. Furfural is more toxic to ethanol fermentation than HMF and other inhibitory molecules due to inhibitions of primary carbon catabolism enzymes, including acetaldehyde dehydrogenase, alcohol dehydrogenase, aldehyde dehydrogenase, glyceraldehydre-3-phosphate dehydrogenase, and pyruvate dehydrogenase [109]. Assimilation of the sulphur-containing amino acids cysteine and methionine were also found to be affected by furan derivative. Furans also correlated to an increase, in reactive oxygen species (ROS), which can damage the mitochondria and vacuole membranes (the cytoskeleton and nuclear chromatin) [110]. On some occasions, furfural is converted to other forms of inhibitory compounds, such as furfuryl alcohol and furoic acid by some yeast species [110]. HMF had been found to be less inhibitory to microbial activity when compared to furfural but can increase the lag phase and deplete the cell growth. It also lasts much longer than furfural because the conversion rate of furfural is 4 times faster than that of HMF, causing microbial process to last longer [111].

### 5.3. Small Organic Acids

Weak organic acids such as acetic, formic, lactic, and levulinic acids are found in the pretreated hydrolysates, which can hinder microbial cell growth. The dissociation form of small organic acids on the cell membrane can lead to an influx into cytosol and improper ion transportation, resulting in inhibited cell growth and productivity [112,113,114]. These kinds of acids can usually be generated from the acetyl groups linked to the sugars or from the hemicellulose backbones during pretreatment, with formation being highly dependent on pretreatment conditions. Minor weak acids such as gallic acid, caproic acid, furoic acid, benzoic acid, and vanillic acid, have also been identified in pretreated hydrolysates [106]. Low molecular weight (MW) organic compounds can be more toxic to microorganisms than high MW compounds and inhibit fermentation. Low MW organic compounds or their salts have be shown to penetrate cell membranes and disrupt the activity of sugar and ion transportation, resulting in growth and performance inhibition [115]. In the case of acetic acid, a common byproduct produced in both hydrolysis and fermentation; it has been reported that yeasts have a tolerance up to roughly 5 g/L concentration of the undissociated forms of acetic acid [116]. Since undissociated forms of carboxylic acids can go through the microbial cell membrane and then decrease the internal pH of the cell, these more significantly affect microorganisms rather than the dissociated forms of the carboxylic acids.

### 5.4. Soluble Sugars

Soluble hydrolysis intermediates and end products of cellulose digestions, such as monomeric sugars and short cellulolignosaccharides, are considered as main contributors to inhibit enzyme activity [117,118]. Several works observed that formation and accumulation of some of these products, including, glucose, cellobiose, and cello-oligomers, inhibited cellulase activity during the enzymatic hydrolysis [119,120]. Further study investigated that β-glucosidase and cellobiohydrolase were shown to be inhibited by glucose and cellobiose, respectively [121]. Cellobiose is one of the most potent inhibitors to cellulase, due to competitive binding with cellulose. The cellobiose binding affinity for cellulase from *Thermomono spora* is 14 times stronger than glucose; 6 times stronger with *T. reesei*; and 3 times stronger with *T. longibrachiatum* [120]. The cellulase from *T. reesei* was also shown to be susceptible to inhibition by cellobiose, glucose, ethanol, butanol, and acetone, and thus is considered a stronger inhibitor [120]. Other studies by Ladisch et al. [122] and Gong et al. [117] confirmed that 0.2–0.4 g/L glucose is inhibitory to cellobiase activity (initially 40 CBU/g cellobiose) by up to 50%.

Recent studies have also demonstrated that hemicellulose products, such as xylose, xylan, and xylo-oligosaccharides, can inhibit enzymatic cellulose hydrolysis. These by-products obstruct the active sites where the enzyme bonds to on cellulose, leading to deactivation of enzyme action on cellulose [47,85,123]. Qing et al. [123] verified that the hydrolysis of defined cellulose (Avicel) at 2% solid concentration combined to 5 FPU/g glucan cellulase and 1.67 mg xylo-oligomers/mL resulted in the 38% lower yield than control test in the absence of xylo-oligomers (81%). However, xylose, xylan, and xylo-oligomers had little to no negative effects on β-glucosidase [123,124]. To overcome the secondary inhibition of hemicellulose degradation molecules (mainly xylo-oligomers), the supplementation of hemicellulase [84,125] or dilute acid treatment [19,86] were proposed to improve the cellulose conversion by removing the adverse effects of xylo-oligomers before cellulase hydrolysis. For example, when xylanase and β-xylosidase were supplemented to the AFEX pretreated corn stover solids before cellulase addition for enzymatic hydrolysis, glucan conversion to glucose was remarkably increased by up to 83% compared to the test without hemicellulase treatment (57% conversion) [125]. It has been thought that the accessary activity of hemicellulase contributed toward reducing the structural hinderance as well as enhaced the cellulase-boosting effect by minimizing non-productive bindings of enzyme by inhibitoty molecules [84,126].

## 6. Strategies to Cope with Inhibition Issues

Cellulolytic enzyme activity and microbial productivity are liable to be inhibited and deactivated by unfavorable degradation products of lignocellulosic materials. In order to counteract inhibitory species, many researches and efforts have been employed to avoid and/or minimize inhibition problems before/after pretreatment process, as briefly summarized in Table 4.

### 6.1. Selection and Modification of Feedstock

Enzyme inhibition due to non-productive binding by small inhibitory molecules such as phenolics, lignin, and lignin degradation products is highly dependent on pretreatment, biomass composition, its chemical structure, and lignin content. Since lignin is strongly recalcitrant in lignocellulose, it needs to be disrupted and removed prior to enzymatic hydrolysis performances. Examination and selection of potential feedstocks with less recalcitrance has been considered as a method to avoid non-cellulose content in mild conditions. For example, *Populus trichocarpa* trees have been suggested to be a suitable biomass for bioethanol production with a less recalcitrance [127]. Lignin syringyl/guaiacyl (S/G) ratio correlated to sugar release from pretreated poplar, with a significant negative impact when the S/G ratio < 2.0. However, this negative effect was reduced when the S/G ratio was higher than 2.0, resulting in a higher sugar yield [127]. Plant genetic engineering has also been explored as a method to change the composition and ratio of lignin S/G to decrease the recalcitrance of syringyl-rich lignin, so that it can be more easily hydrolyzed by LHW pretreatment or maleic acid treatment [129,130]. Fu et al. [142] also observed that genetically modified switchgrass with a lower lignin content could increase the total ethanol yield by up to 38% when compared with the non-modified switchgrass. This study implies that transgenic biomass with low lignin can be pretreated effectively under relatively mild pretreatment conditions and still have effective enzyme loadings [142].

### 6.2. Removal of Inhibitory Compounds

Elimination of inhibitors in the hydrolysate and solid fractions by conditioning or detoxification is the most general method to alleviate the inhibition issues [12,143]. Various conditioning methods have been proposed and tested, including treatment with chemical additives [131], sulfite addition [144], activated carbon treatment [19,41], liquid-liquid extraction [145], and lignin-blocking agents [33,34,41,146,147,148]. The fundamental mechanism of the chemical additives method is to aggregate, precipitate, and/or adsorb the undesirable compounds from the hydrolysates and thereby maintain inhibitors at low effective concentration, which minimizes the inhibitory effects to enzymes and microbes [130,149]. However, the efficiency of this approach depends on various factors, e.g., pretreatment conditions, feedstocks properties, inhibitor concentrations and additive dosage. For instance, the use of bovine serum albumin (BSA) as a lignin-blocking additive was shown to be remarkably effective at improving the enzymatic hydrolysis of pretreated hardwood by decreasing unproductive adsorption of enzymes to other molecules [41]. Pre-culture with BSA at 50 mg/g solids prior to enzyme digestion (3.5 mg enzyme protein/g total solids) resulted in a 90% conversion yield, whereas control (without BSA treatment) only obtained about 30% yield [41]. Further studies of the lignin effect on enzyme activities highlighted that as the enzyme loading was decreased and the ratio of lignin to exposed enzyme was subsequently increased, a noticeable enzyme inhibition was observed, mainly due to non-productive binding of enzyme to the lignin [33]. Also the severity factor of pretreatment is considered to be a major contributor to release the strong lignin and lignin-derived compounds, which could be more severe to enzyme activities. For example, the 1% (*w*/*v*) lignin-free cellulose conversion (Avicel) in the presence of the 0.5% (*w*/*v*) isolated lignin (severity log *R*_0_ = 10.44) at 8 mg enzyme protein/g glucan gave 58% conversion, while when the isolated lignin from higher severity factors of log *R*_0_ = 11.39–12.51 was added, the cellulose conversion to glucose yield was decreased by 51%. Activated carbon also showed removal on inhibitory compounds, binding and/or sequestering of most of furan derivatives, acetic acid, and phenolics in the slurry [19,87]. In particular, activated carbon is able to absorb and/or remove the total phenolics effectively; the recent work showed that the most of the phenolics were reduced from 132 AU to 8 AU after the activated carbon treatment [19]. However, it also has a chemical property that absorbs the soluble pentose and hexose, which causes a loss of fermentable sugars [19,87]. As another attempt, Aghazade et al. [145] demonstrated that liquid-liquid extraction (LLE) approach using ethyl acetate solvent was able to extract 90% acetic acid, which resulted in an 11% higher ethanol yield. Although this method may not be practical in the industry because of the additional solvent supplements and extraction processes, it provides a novel scalable technology and strategy to alleviate the inhibitory compounds in the pretreated lignocellulosic materials. The main challenge with implementing a detoxification approach is that these processes require an additional independent process step that may increase a concern of capital evaluations. A recent techno-economic analysis study reported that the cellulosic ethanol production is currently available around $2.5/gallon [150]. The properties of ethanol production are: simulation with different agricultural feedstocks, acid pretreatment, detoxification with activated carbon, enzyme hydrolysis, fermentation with *Pichia stipites* and *S. cerevisiae*, and distillation [150].

### 6.3. Biological Detoxification

To avoid energy intensive processing conditions, harsh chemicals and expensive processing materials, an environmentally-friendly way to implement a detoxification process, using specific microbes, was also investigated. In this strategy, microorganism pretreatment could alleviate and/or eliminate lignocellulosic-derived inhibitors before enzymatic hydrolysis and subsequent fermentation [84,133,134,151,152]. Several microorganisms, such as *Coniochaeta ligniaria*, *Paecilpmyces variotii*, *Urebacillus thermosphaericus*, and genetically modified *S. cerevisiae* were suggested and evaluated the alleviation of the inhibitors prior to enzyme digestion and microbial fermentation [84,136,144]. Nichols et al. [135] identified *C. ligniaria* NRRL30616 was an ideal candidate as it had increased tolerance to inhibitory compounds and could metabolize these inhibitors (mainly furans and acetate) as a carbon source and energy. The use of *C. ligniaria* was also suitable in the reduction of inhibitors formed during diluted acid pretreatment of different biomass, such as switchgrass, reed canarygrass, alfalfa stem, corn stover, and rice hull, resulting in confirmation ethanol productions with a short lag phase [134,135,136,151]. Especially, the utilization of C5 sugars such as pentose and arabinose in the biologically detoxified hydrolysates with *C. ligniaria* was available to improve the ethanol production using a recombinant bacterium, *Escherichia coli* FBR5. This strain is capable of fermenting both C5 and C6 sugars, but is not preferred to use in the presence of the pretreated hydrolysates due to its sensitivity to inhibitory compounds (furfural, HMF, and acetic acid). However, when the diluted acid pretreated and detoxified corn stover hydrolysates were used as a substrate for microbial fermentation, the FBR5 could effectively consume both C5 and C6 sugars, but could not in the non-biologically detoxified hydrolysates [136]. Another recent example of the detoxification in the liquid hot water pretreated corn stover hydrolysate suggested that the combination of biological detoxification followed by maleic acid or activated charcoal plus enzyme treatment showed the best cellulose conversion to glucose (87%) [19]. However, there are drawbacks with biological treatment; it needs a longer time for additional microbial growth and these additional microbes can have an effect on available sugars. Further investigation is still required for efficient biological detoxification. Recently, the first genome sequence of *C. ligniaria* 30616 was revealed that contained 1070 oxidoreductase, 926 dehydrogenases, 227 decarboxylases, and 23 genes related to oxidative stress. These findings have a strong potential to be applied to genetic and metabolic engineering [153].

### 6.4. Adaptation of Microbial

Other possibilities to counteract with inhibition problems include an adaptive evolution of a selected microbe in medium with inhibitory components, such as hydrolysate samples after pretreatment. This manner was able to develop the microorganism to have a high tolerance to the aliphatic acids, furan aldehydes, and other small organic molecules which had a positive impact on productivity and yield during fermentation [137,138]. An adaptation of a xylose-utilizing genetically modified *S. cerevisiae* strain to steam explosion pretreated sugarcane bagasse hydrolysates resulted in a strain with better fermentation performance than with the non-adapted wild type [25]. This adapted strain showed higher maximum volumetric productivity (0.51 vs. 0.23 g/L/h), maximum specific productivity (2.55 vs. 1.15 g/L/h), and biomass yield on total sugar (0.029 vs. 0.02 g/g) [25]. While a faster fermentation was observed with the adapted strain, the final ethanol yield was similar to non-adapted strain [25]. Similar studies of evolutionary adapted fermenting microorganisms observed the adapted stain was not compatible to other hydrolysates and pretreatment conditions [139,154,155]. More recent study on the microbial adaptation of inhibitors reported that grape marc was a potential source for tolerant yeast strains because it has severe conditions, including limited carbon and nitrogen sources, exposure to solar radiation, low pH, temperature variation between 20 °C and 45 °C, and inhibitory compounds (weak acids, ethanol, and phenols) [156]. The screening and selection of the inhibitor-tolerant and thermostable *S. cerevisiae* strain (Fm13) exhibited great fermentation performances with around 90% of theoretical maximum ethanol yields in the presence of high inhibitor concentrations of acetic acid (7.2 g/L), formic acid (2.44 g/L), lactic acid (6.89 g/L), furfural (2.77 g/L), and HMF (3.75 g/L) [156]. In addition, further microbial fermentation tests in the steam explosion pretreated sugarcane bagasse hydrolysates resulted in similar ethanol yields of 89%, which were 7.7 times higher ethanol production compared to the result from control (benchmark *S. cerevisiae* strain) [156].

### 6.5. Genetic/Metabolic Engineering

Using recombinant strain by genetic/metabolic engineering is widely accepted to overcome the inhibition problems in the fermentation process. Overexpression of specific enzymes, such as alcohol dehydrogenase (ADH) and transaldolase (TAL), in an engineered xylose-fermenting *S. cerevisiae* strain was able to improve the ethanol yield when it was fermented with the high concentration of furfural hydrolysates [157]. In this study, they identified that some genes were significantly related to the pentose phosphate pathway and the modified strain could upregulate to furfural inhibition. Similar studies have been performed on the fungus *Trametes versicolor* and bacteria *Escherichia coli* that have an increased tolerance to spruce wood hydrolysates [139] and sugarcane bagasse hydrolysates [140], respectively. These microbes were proposed as an alternative for ethanol fermentation. Multiple studies have figured out the modified strain was tolerant to targeted inhibitors and enhanced the cell growth and production of ethanol. However, generation of inhibitory compounds are significantly related to lignocellulosic biomass and pretreatment condition, the modified strain may not be appropriate to use as a detoxification methods [94,158].

## 7. Conclusions

Various physico-chemical pretreatment methods for biochemical conversion of lignocellulose materials have been employed and greatly improved, that mainly disrupt complex structure of biomass and remove non-cellulose contents (hemicellulose and lignin), thus promoting cellulose conversion to monomeric sugars. However, the formation of inhibitors during pretreatment and their inhibition problems on enzymes and microbial activities are still limitations that need to be further examined. Recent studies have focused more on better understanding and knowledge on how to alleviate and/or encounter inhibition problems, for example, Wuddineh et al. [159] identified that the *PvKN1* gene, a putative ortholog of maize Knotted1 transcription factor (KN1), could be overexpressed in switchgrass that resulted in increased the scarification yields, by reduced the lignin content and modified the cellulose and hemicellulose biosynthesis. Similar study done by Voorend et al. [160] highlighted the overexpression of GA20-OXIDASE1 was able to affect cell division and cell expansion that enhanced the saccharification efficiency in maize after NaOH pretreatment. Meanwhile, novel strategies to minimize unproductive enzyme bindings on lignin and lignin-derived molecules using BSA [33,34,41], soybean and whey proteins [161], hydrophobic proteins from corn stover [162], and bacterial proteins [87] were recently proposed and developed. In addition, genetic/metabolic engineering of feedstocks for the high-quality biomass, and manipulation of the concerned strains’ pathway appear to be a feasible strategy to improve bioethanol yields without further separating processes. We believe the proper understanding of the lignocellulose recalcitrance and the management of inhibition problems are considered as a rapid process to optimize the cellulosic ethanol production as well as industrially important bioproducts.

## Figures and Tables

**Table 1:** Chemical composition of common lignocellulosic feedstocks (% dry basis).

Biomass	Cellulose	Hemicellulose	Lignin	Reference
Bagasse	39.0	24.4	24.8	[14,15]
Barley hull	33.6	37.2	19.3	[16]
Corn fiber	14.3	16.8	8.4	[17]
Corn pericarp	22.5	23.7	4.7	[18]
Corn stover	37.0	22.7	18.6	[19]
Wheat straw	30.2	21.0	17	[20]
Red maple	41.0	15.0	29.1	[21]
Rice straw	31.1	22.3	13.3	[22]
Rye straw	30.9	21.5	22.1	[23]
Switchgrass	39.5	20.3	17.8	[24]
Sugarcane bagasse	43.1	31.1	11.4	[25]
Sweet sorghum bagasse	27.3	13.1	14.3	[26]
Olive tree pruning	25.0	11.1	16.2	[27]
Poplar	43.8	14.8	29.1	[28]
Pinewood	40.0	28.5	27.7	[29]
Spruce	43.8	6.3	28.3	[30]

**Table 2:** Lignocellulosic biomass structural/chemical properties and their recalcitrant effects on pretreatment and enzymatic hydrolysis.

Biomass Property	Effects on Pretreatment and Enzymatic Hydrolysis	Reference
Cellulose crystallinity	The intramolecular and intermolecular chemical linkages such as hydrogen bonding in the linear cellulose chains increase the feedstock recalcitrance, enzyme loading, and pretreatment severe condition. The high cellulose crystallinity contributes to the feedstock recalcitrance, and subsequently decreases the cellulose conversion.	[37,38,39,40]
Degree of polymerization (DP)	Cellulose DP is normally in the range of 800–10,000 (up to 17,000). Since the high DP structure has less reducing sugar ends that could affect feedstock disobedience and enzyme catalyst, the reduction of DP is required for effective cellulose conversion	[41,42,43,44,45]
Lignin	Lignin plays a key role in the lignocellulosic materials as a biological glue and secondary cell wall. Both lignin and its roles have negative effects on pretreatment, enzyme usage, cellulose conversion, and total costs. Delignification and/or reduction of lignin content using pretreatments, genetic/system engineering, and feedstock selection/modification are required to improve the final conversion yield and productivity.	[33,34,36,46,47,48,49,50,51,52,53]
Hemicellulose	Xyan, the most plentiful hemicellulose in plants, forms a coating layer with cellulose by hydrogen bonding and covalently links with lignin to protect the plant cells. Primary role of the pretreatment is to solubilize the hemicellulose components, and it could improve the cellulose digestibility and hydrolysis.	[54,55,56,57]

**Table 3:** An overview of aqueous soluble inhibitory compounds generated from physico-chemical pretreatment.

Method	Feedstock (Solid Concen.)	Pretreatment Conditions	Soluble Inhibitors in Pre-Hydrolysate (g/L)				Ref.
			Phenols	Furans	Acetic Acid	Others	
Steam explosion	Olive tree pruning (20%)	Temp. 190–240 °C, residence time 5 min, sulfuric acid 0–2%	nm ^1^	0–3.2	0.4–4.2	Formic acid, 0.8–1.8	[27]
Steam explosion	Wheat straw (30%)	Temp. 190–210 °C, residence time 2–10 min, sulfuric acid 0.2%	nm	0.16–2.14	0.04–1.01	nm	[63]
Steam explosion	Wood chip(38–41%)	Temp. 180–210 °C, residence time 4–12 min, sulfuric acid 0.25–0.5%	nm	0.5–3.2	up to 7.5	nm	[61]
LHW	Maple (23%)	Temp. 180–200 °C, residence time 24 min	1.3	4.1	13.1	Sugar oligomer 12.7, xylo-oligomers 11.2	[86]
LHW	Hardwood (15%)	Temp. 195 °C, residence time 10 min	5.9	0.7	2.5	Gluco-oligomers 3.4, xylo-oligomers 56, formic acid 1.9, bound acetyl 12.9	[87]
LHW	Sugarcane bagasse (10%)	Temp. 180–200 °C, residence time 30 min	1.4–2.4	0.5–5.1	1.1–3.4	Gluco-oligomers 0.8, xylo-oligomers 6.5–12.5	[95]
LHW	Corn stover(10–20%)	Temp. 190 °C, residence time 45 min	181–246 AU ^2^	0.74–8.37	2.0–2.8	Xylo-oligomers 9.71–21.7	[19,84]
AFEX	Poplar	Temp. 180 °C, 233% moisture ammonia 1:1, 2:1, and 3:1 *w*/*w* biomass	2.1 mg/g solids	8.6 µg/g solids	nm	Aliphatic acid 1.8 µg/g solids	[97]

nm ^1^: not measured; AU ^2^: Absorbance Unit.

**Table 4:** Summary of strategies to counteract lignocellulose-derived inhibitors released during pretreatment process.

Strategy	Main Effect	Considerations	Reference
Biomass selection and modification	Screen adequate feedstock and/or engineering which produce less undesirable compounds	A range of suitable agricultural residues, requiring time for selection and engineering	[127,128,129,130]
Detoxification/conditioning	Chemical supplementation, i.e., alkaline, BSA, polymers	Chemical needs, additional process may be required	[33,34,131,132,133]
Biological detoxification	Use microbes	Time consuming, loss of sugars	[19,84,85,134,135,136]
Adaptation of microbes	Adaptive evolution of specific microbe in the inhibitory environment	May not be applied to other feedstock, pretreatment conditions	[137,138]
Genetic/metabolic engineering	Use genetically modified microbes to lignocellulosic hydrolysates	Require following the genetically modified micro-organisms (GMM) process	[139,140,141]
